# 171. Efficacy and Immunogenicity of WRSs2, a Live Attenuated Shigella sonnei Vaccine Candidate, to Protect Against Shigellosis After Challenge with a Wild-type S. sonnei strain 53G

**DOI:** 10.1093/ofid/ofaf695.001

**Published:** 2026-01-11

**Authors:** Robert W Frenck, Nadine Rouphael, Shahida Baqar, Michelle Dickey, Veronica Smith, Jill El-Khorazaty, Monica McNeil, Christina M Quigley, Erin Scherer, Jamie Fraser, Tena Pham, Carson Caldwell, Lori Newman, Sarah Bechnak, Shoshana Barnoy, Lakshmi Chandrasekaran, Chad Porter, Susan Heard, Akamol Suvarnapunya, Krista Cato, Malabi Venkatesan

**Affiliations:** Cincinnati Children's Hospital, Cincinnati, OH; Emory University School of Medicine, Atlanta, Georgia; NIH, Rockville, Maryland; Cincinnati Children's Hospital, Cincinnati, OH; Emory University School of Medicine, The Hope Clinic of the Emory Vaccine Center, atlanta, Georgia; Emmes, Rockville, Maryland; Cincinnati Children's Hospital, Cincinnati, OH; Cincinnati Children's Hospital Medical Center, Cincinnati, Ohio; Emory University School of Medicine, Atlanta, Georgia; Emmes, Rockville, Maryland; Cincinnati Children's Hospital, Cincinnati, OH; Cincinnati Children's Hospital, Cincinnati, OH; NIH, Rockville, Maryland; Emory University, atlanta, Georgia; Walter Reed Army Institute of Research, Silver Spring, Maryland; Walter Reed Army Institute of Research, Silver Spring, Maryland; Naval Medical Research Center, Silver Spring, Maryland; Emmes, Rockville, Maryland; Walter Reed Army Institute of Research, Silver Spring, Maryland; NIH, Rockville, Maryland; Walter Reed Army Institue of Research, Silver Spring, Maryland

## Abstract

**Background:**

*Shigella* is a leading cause of bacterial diarrhea. The live attenuated *S. sonnei* vaccine candidate WRSs2, lacking virG (icsA), senA, and senB, was evaluated for its efficacy in preventing shigellosis.

**Figure:**
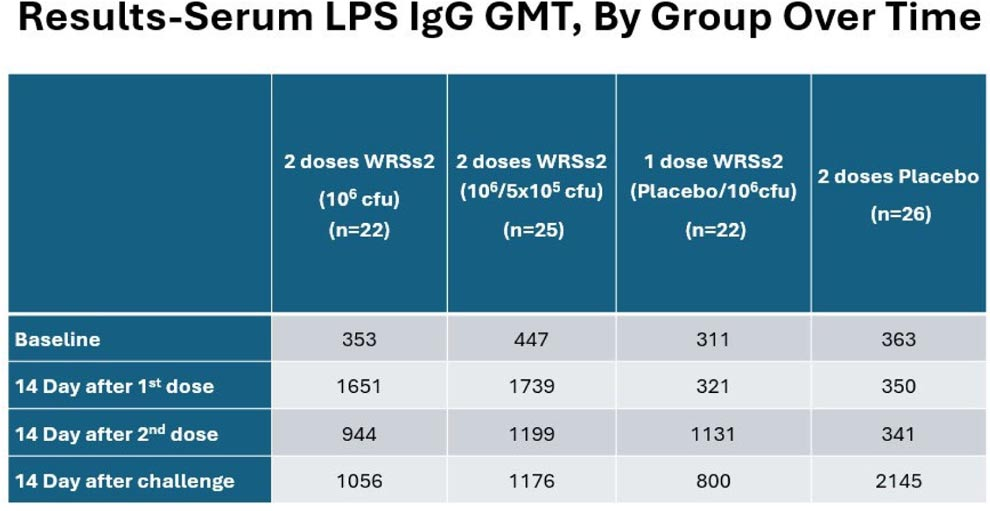
Serum LPS IgG GMT, By Group Over Time

**Figure:**
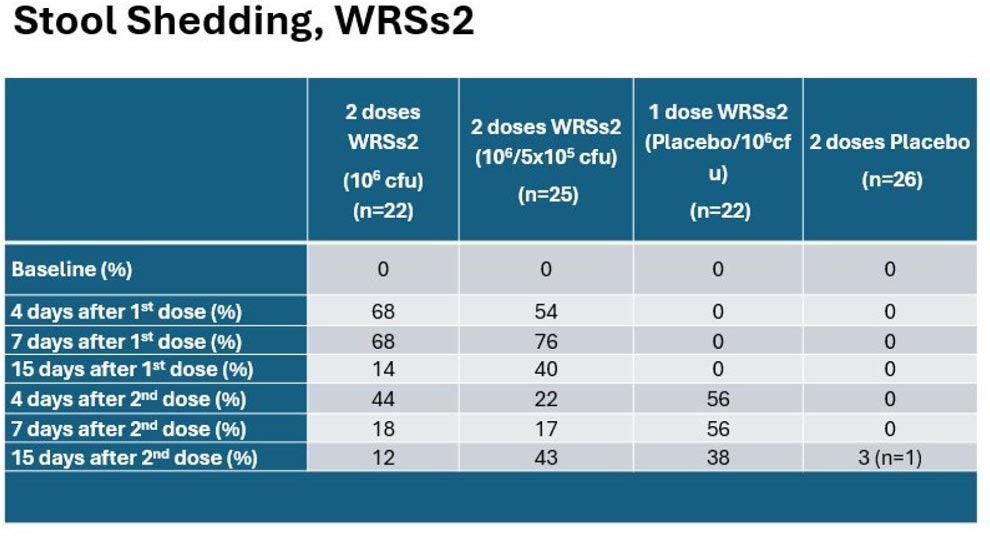
Stool Shedding, WRSs2

**Methods:**

Adults aged 18-49 years received 2 doses of study product 28 days apart (1 or 2 doses of 10^6^ colony forming units (cfu) of WRSs2 or placebo). 4 weeks after vaccination, using a controlled human infection model (CHIM), participants were administered 1500 cfu of virulent S. *sonnei* (53G) in an inpatient unit. After challenge, participants were evaluated daily and stools were graded and cultured for *Shigella*. 5 days post-challenge, ciprofloxacin (500 mg BID x 3 days) was administered. Discharge home was based on antibiotic completion and 2 stool cultures negative for *S. sonnei*. Blood samples were collected at various times after each vaccination as well as after challenge. Shigellosis was defined as signs and symptoms of Shigella along with Shigella isolated from the stool. Vaccine efficacy was calculated as 1 – (attack rate of shigellosis in WRSs2 group /attack rate of shigellosis in placebo group).

**Figure:**
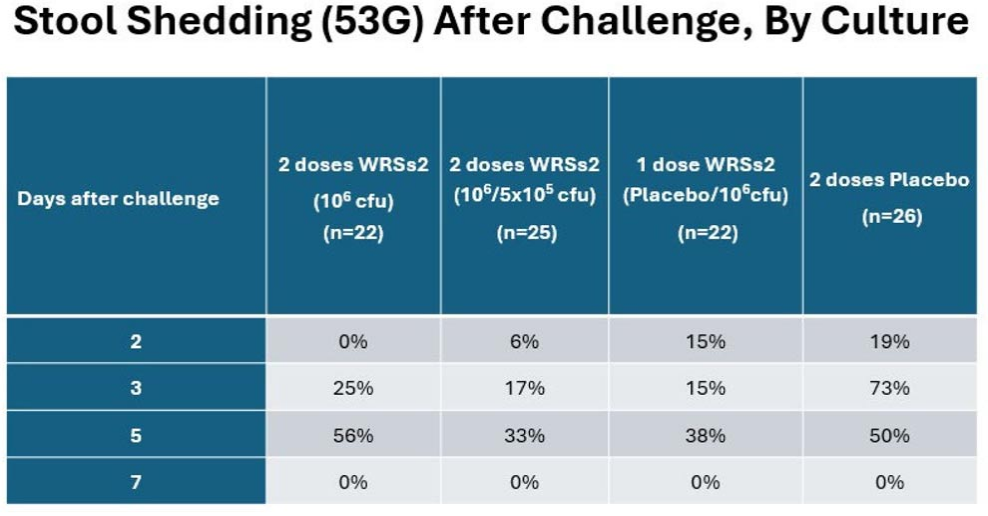
Stool Shedding (53G) After Challenge, By Culture

**Figure:**
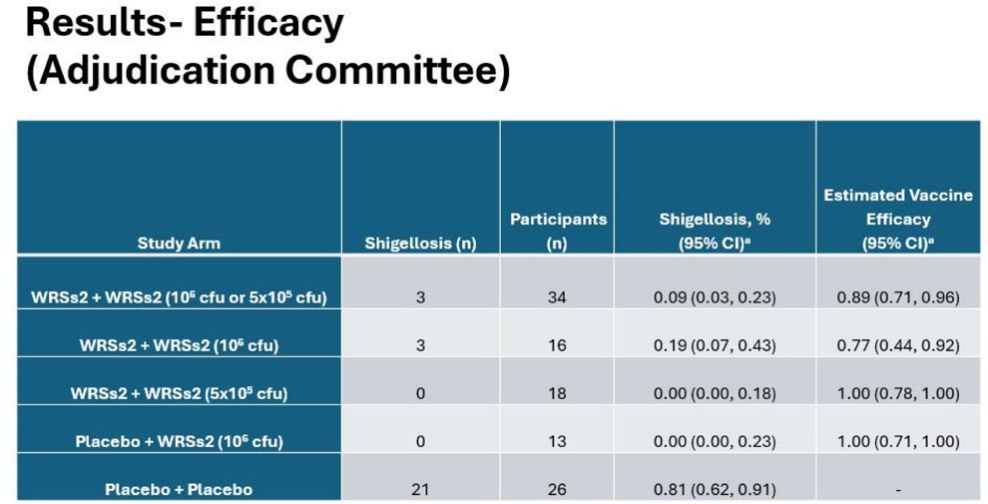
Efficacy of WRSs2 in Prevention of Shigellosis After Challenge with 53G

**Results:**

108 participants were randomized to receive 1 or 2 doses of WRSs2 (n=23 and n=48 respectively) or 2 doses of placebo (n=37). 73 participants (13 one-dose WRSs2, 34 two-dose WRSs2, 26 placebo) received a dose of 53G. The vaccine was generally well tolerated. 2 participants developed Grade 3 diarrhea with no alternative etiology resulting in a vaccine dose reduction to 5x10^5^ cfu and limiting remaining enrollments to two arms (2 doses WRSs2 vs. 2 doses placebo).

Among participants receiving 2 doses of WRSs2, vaccine efficacy was 89% (95% CI: 71-96%), p< 0.001 (9% shigellosis in vaccinees vs 81% in placebo recipients). No participant in the 1 dose WRSs2 group developed shigellosis (VE=100% (95% CI: 71-100%)).

Following vaccination, most participants mounted serum IgA and IgG responses to Invaplex and LPS. The responder rates to these antigen or isotypes among vaccine recipients varied from 45-73% vs 0-6% among placebo recipients.

**Conclusion:**

Using a *S. sonnei* CHIM, the live attenuated oral *S. sonnei* vaccine WRSs2 demonstrated high efficacy against shigellosis. These findings support further evaluation of WRSs2 in field trials to assess its effectiveness in endemic settings.

**Disclosures:**

All Authors: No reported disclosures

